# Socio-ecological dynamics of disease transmission in rewilding landscapes: A case study of the Northern Drakensberg Nature Reserve, South Africa

**DOI:** 10.1016/j.onehlt.2026.101653

**Published:** 2026-11-06

**Authors:** Samuel Akpan, Helen Esser, Rebekka Assink, Laxmi Chaulagain, Frank van Langevelde, Percy Moyo, Gilian van Duijvendijk, Mbongiseni Shongwe, Kirsten Oliver, Claudius van de Vijver, Arash Ghoddousi

**Affiliations:** a Wildlife Ecology and Conservation Group, Wageningen University & Research, Droevendaalsesteeg 3A, 6708 PB Wageningen, the Netherlands; b Institute of Genomics & Global Health (IGH), Redeemers University, PMB 230, Ede, Nigeria; c International Livestock Research Institute (ILRI), PO Box 30709, GPO, 00100 Nairobi, Kenya; d Soil Geography & Landscape Group, Wageningen University & Research, Droevendaalsesteeg 3, 6708 PB Wageningen, the Netherlands; e Wildlands Conservation Trust, PO Box 21450, Mayors Walk, 3208 Pietermaritzburg, South Africa; f Wageningen Environmental Research, Wageningen University & Research, PO Box 47, 6700 AA Wageningen, the Netherlands; g Production Ecology and Resource Conservation (PE&RC) Graduate School, Wageningen University, Wageningen, the Netherlands

**Keywords:** Disease risk, Wildlife re-introduction, Conservation, Human-livestock-wildlife interface, Pathogen exposure, One Health

## Abstract

Rewilding is increasingly promoted as a conservation strategy for restoring biodiversity and ecosystem processes. Yet rewilding efforts could involve species (re-)introductions that have implications for infectious disease dynamics. Despite the importance of such socio-ecological dynamics at the human-livestock-wildlife interface, the role of rewilding in disease prevalence remains poorly understood. We conducted semi-structured key informant interviews and focus group discussions with 24 stakeholders in the Northern Drakensberg Nature Reserve, South Africa, to examine how ecological dynamics, livestock management practices, governance conditions, and stakeholder perceptions shape disease transmission risks in a rewilding landscape. We analyzed the data using inductive thematic analysis guided by the One Health framework. Ecological and governance factors revealed that scavenger species, particularly vultures, jackals, and free-roaming dogs, shaped carcass persistence and pathogen exposure pathways, while the absence of coordinated governance across private landowners, conservation authorities, park managers, and community members created major gaps in disease surveillance and response. Disease risks were rarely considered in wildlife introduction decisions. Conservation authorities often assumed that pre-introduction health screening was sufficient, with limited attention given to post-release ecological dynamics. Disease awareness varied considerably across stakeholder groups and was influenced more by livelihood practices, concerns over grazing competition, and human safety than by epidemiological understanding. Historical exclusion from conservation decision-making further reinforced distrust, undermining disease reporting and compliance with interventions. These findings demonstrate that disease risks in rewilding landscapes emerge through interacting ecological, behavioural, and institutional processes rather than wildlife dynamics alone. Integrating diverse stakeholder perspectives into rewilding governance will be critical for operationalizing One Health approaches and anticipating unintended health consequences.

## Highlights

• Livelihood, safety, and livestock grazing concerns overshadow stakeholders' perceptions of disease risks.
• Scavengers (vultures, jackals, etc) are key ecological modulators of disease risks.
• Reinforcement of herbivore populations increases opportunities for wildlife-livestock spatial overlap.
• Restoration of apex predators can reduce zoonotic risks through top-down regulation of reservoir host populations.
• Disease risks arise from socio-ecological and institutional interactions rather than through biological interfaces alone.

## 1 Introduction

Rewilding has gained increasing prominence as a conservation strategy aimed at restoring ecosystem processes, biodiversity, and trophic complexity. It does so through the (re)introduction or reinforcement of wildlife species and the reduction of human intervention [1]. Advocated to reverse biodiversity loss and enhance ecosystem resilience, rewilding initiatives are expanding across diverse ecological and socio-political settings [2]. However, alongside its ecological benefits, rewilding may also generate unintended socio-ecological consequences. The return or reinforcement of wildlife populations can increase human-wildlife conflict, including livestock depredation, threats to human safety, crop damage, and infrastructure destruction [3]. These impacts can impose substantial economic burdens on rural communities, reshape land-use practices, and influence attitudes toward conservation [4].

Rewilding may also alter species interactions, with consequences for scavenger dynamics, resource competition, and patterns of animal movement [5]. For instance, the reintroduction of large predators has been shown to alter ungulate behaviour, subsidize scavengers, and modify disease dynamics [5]. Such changes can, in turn, reshape infectious disease dynamics by modifying host communities, contact rates, and transmission pathways at the human-livestock-wildlife interface [6]. Despite the importance of disease transmission to socio-ecological wellbeing, this aspect has rarely been considered in the context of rewilding initiatives [7].

Rewilding has often been discussed as a potential way of reducing zoonotic risk, because restoring ecosystems may alter infectious disease dynamics. For example, van Volkenburg et al. [8] emphasize that restoring trophic complexity and ecological integrity may regulate reservoir host populations, reduce pathogen amplification, and strengthen natural disease control mechanisms. However, this perspective largely highlights rewilding's potential protective effects, with comparatively little attention to contexts in which rewilding may also introduce new disease risks or reshape transmission pathways. As a result, the possibility that rewilding could amplify disease risk remains insufficiently examined, particularly in complex human-dominated landscapes. Such amplification could occur through altered host assemblages, increased wildlife-livestock contact, or socio-economic and governance constraints.

Ecological factors alone, however, do not determine disease risk. There is growing recognition that it is also influenced by social, economic, and institutional processes that determine how humans interact with animals and respond to disease threats [9]. Yet the role of such contextual factors in disease dynamics in rewilding landscapes remains less understood.

Governance arrangements, livelihood practices, risk perception, and access to veterinary and health services all influence how disease risks emerge and are managed in rewilding landscapes [10], [11], [12]. A lack of coordination between the conservation and health sectors creates governance challenges. At the same time, livelihood practices such as communal grazing and livestock movement across shared grazing areas increase wildlife-livestock contact [6].

The One Health framework, defined as a collaborative, multisectoral, and transdisciplinary approach that recognizes the interdependence of human, animal, and environmental health [13], provides a conceptual foundation for integrating these factors when assessing disease risk in rewilding landscapes. Although the framework is gradually gaining acceptance in policymaking and research [14], in practice it has largely been operationalized through technical interventions, such as pre-release pathogen screening of translocated animals, vaccination campaigns, and diagnostic surveillance [7], [8]. Far less attention has been paid to how stakeholders perceive and navigate disease risk in their everyday interactions with wildlife and livestock [8]. Qualitative research can help fill this gap by showing how disease risk is understood, prioritized, and acted upon across stakeholder groups [15].

Despite Africa being central to global debates on conservation, wildlife-livestock disease dynamics, and zoonotic spillover, empirical studies examining rewilding through a socio-ecological disease lens remain scarce [12], [16]. This gap is particularly relevant in countries like South Africa, where protected areas are often embedded directly within livestock-dependent communities. High wildlife host diversity, frequent wildlife-livestock overlap, and endemic zoonotic and livestock diseases such as bovine tuberculosis and brucellosis create conditions conducive to cross-species transmission [17], [18]. These ecological risks intersect with socio-economic inequalities, limited access to veterinary services, and historical exclusion from conservation governance, thereby constraining disease surveillance and control [10], [12]. Understanding disease dynamics in such contexts is therefore critical for assessing the health implications of rewilding.

The Northern Drakensberg Nature Reserve (NDNR) in KwaZulu-Natal, South Africa, provides a valuable case study for examining the rewilding-disease nexus. Ongoing conservation efforts, adjacent communal and commercial livestock systems, and diverse stakeholder interests characterize the reserve. The reserve was formally established through a collaborative conservation initiative that aims to integrate biodiversity protection with local socio-economic activities, including livestock and agricultural land uses [18], [19]. Conservation initiatives within the reserve and surrounding areas include ecological restoration, reinforcing native wildlife populations, protecting scavenger species such as vultures, and management interventions to restore ecosystem function and biodiversity. These efforts reflect broader rewilding objectives focused on restoring ecological processes, enhancing wildlife populations, and reducing anthropogenic pressures.

Despite growing interest in rewilding and zoonotic disease risk, few studies have empirically examined how stakeholders perceive, construct, and govern disease risk in African rewilding landscapes. Most have focused instead on the ecological outcomes of wildlife reintroduction rather than on the social and institutional dimensions of disease governance [7], [12]. Existing research has largely emphasized ecological mechanisms such as host-pathogen dynamics and transmission pathways, with comparatively limited attention to the social, behavioural, and institutional processes that shape disease surveillance, reporting, and control [6], [8]. Addressing this gap is critical for understanding how disease risks emerge and are managed in complex socio-ecological systems.

This study, therefore, examines the socio-ecological and governance dimensions of disease risk in a typical rewilding landscape, using a qualitative One Health approach. We use One Health as an analytical lens: a way of examining how disease transmission risks in rewilding landscapes emerge from the interaction of ecological dynamics, social practices, and governance arrangements. We do not treat it as a normative policy objective, nor do we assume that coordination between sectors will by itself reduce disease risk.

Specifically, we sought to answer the following research questions: (i) What ecological dynamics, livestock management practices, and governance conditions shape disease transmission in a rewilding landscape? (ii) How do stakeholders perceive disease risks associated with rewilding, and how do these perceptions influence disease surveillance, prevention, and response?

## 2 Materials and methods

### 2.1 Study area

The Northern Drakensberg Nature Reserve (NDNR) forms part of the Maloti-Drakensberg Transfrontier Conservation Area, a large transboundary conservation landscape spanning South Africa and Lesotho (Fig. 1). The region comprises protected areas, private game reserves, and adjacent communal lands used for livestock grazing and subsistence agriculture, creating a heterogeneous socio-ecological landscape with frequent spatial overlap between wildlife, livestock, and human activities. The landscape is characterized by montane grasslands, steep escarpments, and marked seasonal climatic variability, with cooler temperatures than surrounding lowland regions [20]. These grasslands support diverse wildlife populations, including large herbivores such as common eland (*Tragelaphus oryx*), as well as smaller ungulates, mesocarnivores, and scavenger species, whose distributions are influenced by land use, grazing intensity, and conservation management [21], [22]. Livestock production, particularly cattle, sheep, and goats, is a dominant livelihood activity in surrounding communal areas, resulting in frequent wildlife-livestock interactions across shared grazing areas and porous conservation boundaries.

**Fig. 1:**
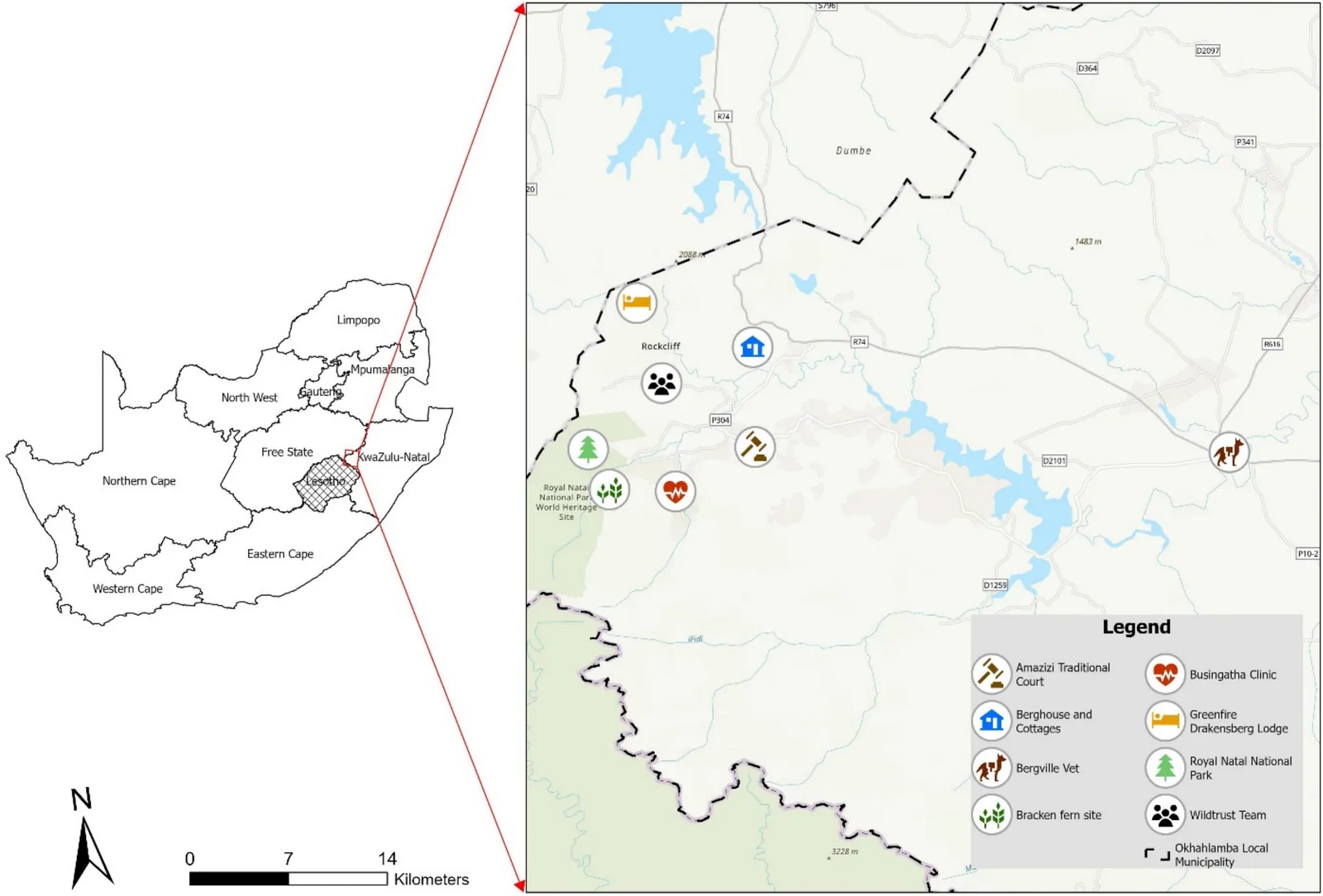
A map of the study area in South Africa (left), and a detailed map showing data collection sites (right).

### 2.2 Study design

We adopted a qualitative case study design, guided by the One Health framework: an integrative analytical approach to examine interactions among human, animal, and environmental health domains and their implications for disease risk [11], [13]. In this study, we operationalized One Health by examining three interconnected components: (i) animal health and ecological dynamics, including wildlife presence, livestock management practices, scavenger activity, and patterns of animal interaction; (ii) human health and behavioural factors, including livestock keeping practices, disease awareness, vaccination behaviour, and healthcare access; and (iii) institutional and environmental governance, including conservation policies, veterinary service provision, surveillance systems, and stakeholder engagement. These components informed the design of interview and focus group guides, which explored how ecological interactions, human practices, and governance arrangements intersect to influence disease risk. During analysis, the framework was used to organize and interpret themes across these domains, allowing examination of how disease risks emerge through interconnected ecological processes, human behaviours, and institutional conditions, rather than through biological factors alone [23].

### 2.3 Participants' selection and recruitment

We selected the participants using purposive sampling to capture perspectives from stakeholder groups directly involved in or affected by rewilding, livestock management, conservation, and health in the Northern Drakensberg landscape. Stakeholder categories included conservation authorities, park management, veterinarians and veterinary suppliers, healthcare workers, private landowners, non-governmental organizations, and community members engaged in livestock keeping and natural resource use. The participant selection aimed to maximize heterogeneity of perspectives rather than statistical representativeness. We identified the initial participants through institutional affiliations and local networks, and recruited additional participants through referral, where appropriate. Sampling continued until thematic saturation was reached, defined as the point at which no substantive new themes emerged during data collection [24]. We assigned anonymized identification codes to each participant to preserve confidentiality while demonstrating representation across stakeholder groups. Codes consisted of a stakeholder group prefix and participant number (e.g., CONS-02 for conservation professionals, VET-03 for veterinary stakeholders, COMM-04 for community members, HCW-01 for healthcare workers, PKM-01 for park managers, CONE-01 for conservation ecologists, and ACAD-01 for non-governmental organization and academia stakeholders.

### 2.4 Data collection

We collected data in March 2025 through semi-structured key informant interviews and focus group discussions, using complementary qualitative methods for exploring individual expertise and shared community perspectives. We conducted one-on-one key informant interviews with participants selected based on their professional expertise, institutional roles, or direct involvement in wildlife management, livestock health, conservation, or healthcare, enabling in-depth exploration of their knowledge, experiences, and institutional perspectives on disease risks and rewilding [25]. The interviews lasted approximately 30–45 min and involved a single participant and one or two researchers [25]. We primarily conducted the interviews in English and interpreted them by a team member fluent in *Zulu*, the local language. The interviews conducted in *Zulu* were translated into English during transcription. To preserve analytic meaning, we reviewed the translations by bilingual members of the research team, with particular attention to idiomatic expressions and culturally embedded terms. We verified ambiguous meanings through discussions within the team and, where necessary, clarified interpretations with local collaborators to ensure contextual accuracy. We conducted focus group discussions with community members engaged in livestock keeping and natural resource use, capturing shared experiences, community-level practices, and collective perceptions of wildlife, livestock management, and disease risks [26]. Each discussion lasted approximately 40–60 min and included 4–8 participants per group [26]. We used protocols (Supplement 1) designed to explore wildlife-livestock interactions, disease awareness, rewilding practices, and governance arrangements [25], [27]. Initial question domains were informed by One Health and socio-ecological disease frameworks, which emphasize the interconnected roles of wildlife, livestock, human behaviour, and governance in shaping disease risk [10], [13], [23]. Questions were deliberately open-ended to allow participants to define disease risks and rewilding implications in their own terms.

We obtained free, prior, and informed consent from all participants after explaining the study objectives, procedures, voluntary nature of participation, and confidentiality protections. Participants had the opportunity to ask questions and could decline participation or withdraw at any time without consequence. We took detailed field notes during all sessions to document contextual observations, non-verbal cues, and emerging insights. We also transcribed audio recordings verbatim and integrated field notes into the transcripts to enhance completeness and preserve contextual meaning.

### 2.5 Ethical considerations

Ethical approval was not required for this study. Verbal consent was obtained from the participants prior to commencement of the study, and this was witnessed by the lead author.

### 2.6 Scenario development

We developed conceptual scenarios as an interpretive analytical tool to synthesize stakeholder narratives and thematic findings [27]. These scenarios do not represent epidemiological reconstructions of specific transmission events. Instead, they integrate themes related to ecological interactions, human behaviour, and governance conditions to illustrate how stakeholders perceived disease risks as evolving within the rewilding landscape. We constructed scenarios by mapping directional influence pathways between themes, using boxes and arrows to represent stakeholder-reported and literature-informed relationships across the three One Health domains. Governance and behavioural modifiers were identified and represented as amplifying or attenuating factors within each pathway. These diagrams are intended as analytical heuristics rather than formal causal loop diagrams or epidemiological models. These scenarios do not estimate disease burden or establish causal transmission pathways. Rather, they provide a structured representation of how stakeholders understood, experienced, and governed disease risk, highlighting perceived pathways through which ecological restoration, scavenger dynamics, livestock management, and institutional factors may influence it.

### 2.7 Data analysis

We analyzed the data using inductive thematic analysis, a qualitative method for identifying, analyzing, and interpreting patterns of meaning (themes) within textual data [28]. This approach allows themes to emerge from the data rather than imposing predefined categories, making it particularly suitable for exploring complex socio-ecological and institutional processes. We followed an iterative, multi-stage process beginning with repeated reading of transcripts to become familiar with the data. We then generated initial codes inductively to capture meaningful features related to disease perceptions, wildlife-livestock interactions, rewilding practices, and governance dynamics. We grouped related codes into broader themes and refined them through constant comparison across participants and stakeholder groups. Finally, we interpreted themes in relation to the One Health framework to examine how ecological, behavioural, and institutional factors interact to shape disease risk in the rewilding landscape.

We generated initial codes inductively to capture meaningful units of text reflecting participants' experiences, observations, and interpretations of disease risk, wildlife-livestock interactions, rewilding practices, and institutional processes [27]. Although coding remained data-driven, we organised emerging codes in relation to the One Health framework, which conceptualizes disease risk as arising from interactions among animal-ecological, human behavioural, and governance domains [13], [23]. For example, codes related to wildlife presence, scavenger activity, and livestock grazing were grouped under animal-ecological interactions, while codes relating to vaccination practices, disease reporting, and livestock management were categorized under human behavioural factors. Codes relating to veterinary access, conservation policies, and stakeholder engagement were grouped under governance and institutional processes. We iteratively refined these categories and grouped related codes into broader themes that reflected how ecological, behavioural, and institutional factors interacted to shape disease risk in the rewilding landscape [28].

Several strategies were employed to enhance the reliability of the findings. Triangulation across stakeholder groups allowed validation of themes from multiple perspectives. The use of verbatim quotations ensures transparency in the link between data and interpretation. Reflexivity was incorporated throughout the research process [29]. Reflexivity refers to the process by which researchers actively deliberate on their assumptions, perspectives, and potential biases, and consider how these may shape interactions with participants and the interpretation of findings [30]. The research team comprised scholars with backgrounds in conservation science, public health, veterinary science, and social research, and included both locally embedded and externally based researchers. This interdisciplinary composition shaped data interpretation by bringing multiple analytical perspectives to the same material. Reflexive discussions were used to interrogate assumptions, particularly around conservation governance, disease narratives, and community knowledge, and to minimize the influence of disciplinary or institutional bias on theme development.

## 3 Results

### 3.1 Participants' characteristics

A total of twenty-four (*n* = 24) participants contributed to the study through key informant interviews and focus group discussions (Table 1). Stakeholders varied markedly in their degree of engagement with wildlife, livestock, and disease management, shaping how disease risks associated with rewilding were perceived and prioritized. Conservation professionals and veterinarians had long-term experience in the region (7 to >30 years), whereas community participants primarily engaged with vegetation management, livestock, or subsistence activities and reported limited involvement in conservation decision-making (Table 1).

**Table 1:** Stakeholder groups involved in the study, their roles in the socio-ecological system of the Northern Drakensberg Nature Reserve, and the number of participants per group contributing through key informant interviews and focus group discussions.

Stakeholder group	Role in the socio-ecological system	Number participating
Conservation authorities	Wildlife management, rewilding, scavenger conservation	4
Park management	Protected area operations	2
Veterinarians & veterinary suppliers	Livestock health and disease control	6
Healthcare workers	Human health and zoonotic disease response	2
Private landowners	Wildlife and livestock management	3
NGOs and academia	Community engagement, research	3
Community members	Livestock keeping, natural resource use	4
**Total**		24

### 3.2 Reported diseases

Participants identified a wide range of diseases affecting livestock, wildlife, domestic dogs, and, to a lesser extent, humans (Table 2). Local names used by participants are given with their causative agents in the table: “wireworm” refers to the barber's pole worm (*Haemonchus contortus*), and *snotsiekte* refers to wildebeest-associated malignant catarrhal fever.

**Table 2:** Diseases reported within and around the Northern Drakensberg Nature Reserve by stakeholders. Causative agents are given in parentheses. Rabies and bovine tuberculosis (BTB) are repeated in the last row because stakeholders perceived them as zoonotic (i.e., affecting humans); both also affect the animal hosts listed in the first row, and the last row does not imply that they affect humans only.

Disease category	Diseases reported	Affected hosts
Directly transmitted	Rabies (rabies virus), foot-and-mouth disease (FMD virus), brucellosis (*Brucella* spp.), bovine tuberculosis (*Mycobacterium bovis*), lumpy skin disease (lumpy skin disease virus), anthrax (*Bacillus anthracis*), *snotsiekte* (wildebeest-associated malignant catarrhal fever; Alcelaphine herpesvirus 1)	Livestock, dogs, wildlife, humans
Vector-borne	Babesiosis (*Babesia* spp.; “redwater”), anaplasmosis (*Anaplasma marginale*; “gallsickness”), heartwater (*Ehrlichia ruminantium*), bluetongue (bluetongue virus), African horse sickness (African horse sickness virus), bovine ephemeral fever (bovine ephemeral fever virus)	Livestock, horses
Parasitic	Wireworm (barber's pole worm, *Haemonchus contortus*), tick infestation (Ixodid ticks)	Livestock
Diseases perceived as zoonotic	Rabies, bovine tuberculosis (BTB)	Humans (in addition to the animal hosts above)

Rabies emerged as the most consistently recognized zoonotic disease across stakeholder groups. Community members frequently associated rabies with domestic dogs, while veterinarians and conservation professionals acknowledged its presence in jackals (*Lupulella* spp.) and other wildlife species:

*“Rabies does occur here, in dogs, vervet monkeys, and jackals. There are vaccination programs, and there is education on rabies, so people should know.”*

(CONS-02)

However, perceptions of other zoonotic risks were mixed. Some health professionals acknowledged limited awareness of livestock-associated zoonoses:

*“I was not aware that tuberculosis can be transmitted from cattle to humans.”*

(HCW-01)

Tick-borne diseases were perceived differently among stakeholders. Park management and conservation staff described ticks as a minor issue due to cooler temperatures, whereas veterinarians and private landowners considered tick-borne infections to be common and economically important:

*“Ticks are a big problem. Redwater, gallsickness, heartwater - we deal with these animal diseases all the time.”*

(VET-03)

### 3.3 Animal-ecological interactions and disease pathways

Within the animal-ecological domain of the One Health framework, rewilding activities were perceived to reshape host assemblages, scavenger communities, and contact rates between wildlife and livestock, thereby influencing the risk of disease transmission. Wildlife introductions and the reinforcement of herbivore populations were understood to increase biomass and carcass availability, with downstream consequences for scavenger dynamics and pathogen exposure.

Scavenger species were identified as key mediators of carcass persistence and disease exposure pathways. Obligate scavengers such as vultures were perceived to facilitate rapid carcass removal, thereby limiting environmental pathogen persistence:

*“Vultures clean carcasses very quickly, which helps limit disease.”*

(CONE-01)

In contrast, facultative scavengers such as jackals and free-roaming dogs were perceived to increase pathogen transmission risk through repeated contact with infected tissues. Participants also noted that different scavenger species access carcasses at different times, creating distinct windows of pathogen exposure:

*“Bearded vultures eat during the day, dogs and jackals come at night.”*

(CONE-02)

Declines in vulture populations due to poisoning were perceived to prolong carcass persistence, increasing contact between infected material and domestic dogs which serve as important rabies reservoirs. Jackals were widely disliked by community members and landowners due to livestock depredation and perceived disease risks:

*“Everyone hates jackals because they kill livestock.”*

(CONE-03)

Across stakeholder groups, rewilding was generally viewed positively in relation to biodiversity restoration and tourism, but disease risk was consistently treated as secondary to habitat suitability, grazing competition, and human safety from wildlife (Table 3).

**Table 3:** Perceptions of rewilding and associated disease risks among key stakeholder groups in the Northern Drakensberg Nature Reserve, including their overall views on rewilding, primary concerns, and perceived disease risks.

Stakeholder groups	Overall view on rewilding	Key concerns	Perceived disease risks
Local community	Positive for non-predatory species	Safety (carnivores), grazing competition	Low; introductions may cause adaptation stress, but not major transmission
Private landowners	Mixed/negative for certain species	No wildebeest, habitat suitability	High for tick-borne and *snotsiekte*
Government/park managers	Positive	Occurrence of diseases	Minimal if checked; large area dilutes density/risks
Veterinarians & veterinary suppliers	Mixed; cautious	Adequacy of disease screening; livestock economic losses	High for tick-borne diseases, brucellosis, and rabies; oppose untested introductions; pre-introduction screening considered insufficient
NGO/academia	Positive	Conservation benefits	Competition > disease; seasonal interactions possible
Health professionals	Neutral	N/A	Low direct impact from reserve

Park managers in particular assumed pre-introduction health screening was sufficient:

*“The main risk is whether this area provides suitable habitat. Diseases are not really considered a risk because animals will be checked beforehand.”*

(PKM-01)

Similarly, community members expressed conditional support for rewilding, with opposition focused almost exclusively on carnivores:

*“You can introduce whatever animal you want, as long as it's not a predator.”*

(COMM-01)

Private landowners, however, were more disease-conscious, selectively opposing the introduction of wildebeest (*Connochaetes taurinus*) due to the perceived risk of malignant catarrhal fever:

*“No wildebeest. Snotsiekte is a real problem, even if the animals are supposedly clean.”*

(PLO-01)

### 3.4 Human behavioural factors

Within the human behavioural domain, disease risk awareness varied substantially across stakeholder groups, shaped by livelihood dependence, institutional roles, and day-to-day interactions with wildlife and livestock systems. Community participants, whose daily priorities centred on livestock and economic conditions, frequently reported limited awareness of wildlife-associated diseases and prioritized immediate livelihood challenges over infectious disease risks:

*“We have no idea about diseases associated with wildlife.”*

(COMM-04)

Several participants explicitly framed disease as a low-priority concern relative to socio-economic pressures such as grazing access, poverty, and livestock theft:

*“Theft is the biggest issue for livestock farmers, not diseases.”*

(ACAD-01)

In some cases, disease perceptions reflected deep distrust of biomedical and institutional narratives, rooted in historical marginalization. One community respondent described rabies as externally imposed:

*“Rabies, like all diseases, is a conspiracy. Developed countries manufacture these diseases and then sell vaccines to us.”*

(COMM-02)

Others attributed rabies to dog management practices rather than wildlife interactions:

*“Our dogs are free to roam and eat whatever is available. That is what causes rabies.”*

(COMM-03)

Livestock management practices further shaped disease transmission pathways. Communal grazing, seasonal movement of livestock into mountainous areas during winter, and communal treatment points where animals are immersed in acaricide solutions to control ticks were all identified as key behavioural drivers of disease risk. Although essential for tick control, shared dipping facilities bring animals from different herds into proximity, creating opportunities for direct disease transmission. Limited fencing further facilitated frequent livestock-livestock and occasional livestock-wildlife contact (Fig. 2).

**Fig. 2:**
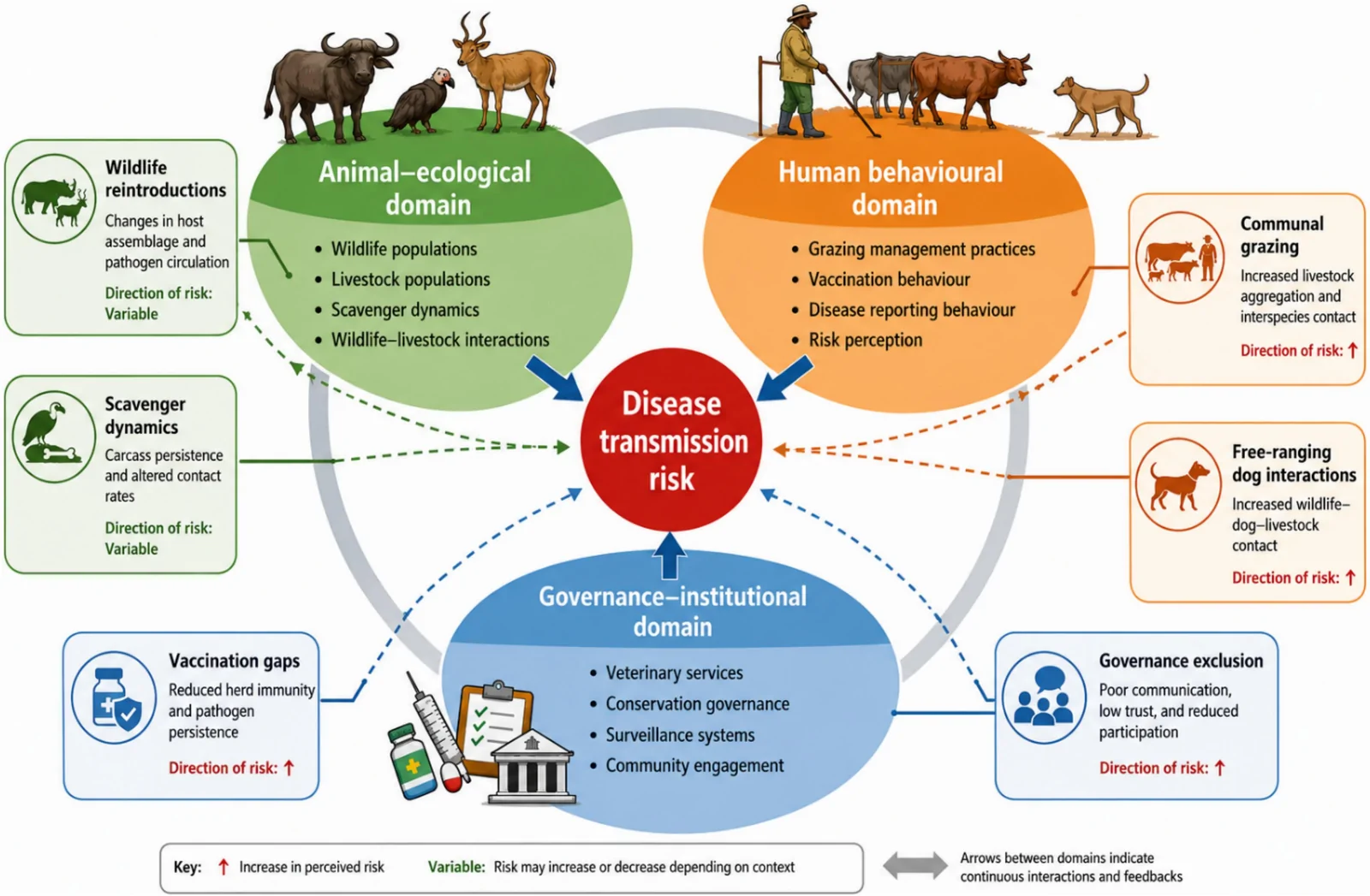
Conceptual illustration of socio-ecological pathways linking rewilding and disease transmission risk in the Northern Drakensberg Nature Reserve, South Africa. The figure shows how interactions across three One Health domains shape disease transmission risk. Peripheral boxes highlight key drivers within each domain, including wildlife reintroductions, scavenger dynamics, communal grazing, free-ranging dog interactions, vaccination gaps, and governance exclusion. Solid arrows indicate direct pathways of influence on the risk of disease transmission. Arrows between domains indicate continuous interactions and feedback. Direction of risk indicators reflects stakeholder perceptions, where upward arrows denote increased risk and “variable” denotes context-dependent effects that may either increase or decrease risk depending on ecological and management conditions.

Community members described efforts to limit disease spread through practices such as isolating sick animals:

*“If a cow gets sick, we isolate it in the camp so we can monitor it.”*

(COMM-03)

However, access to veterinary services, vaccines, and laboratory diagnostics was constrained by cost, distance, and infrastructure, limiting the effectiveness of these behavioural responses.

### 3.5 Governance and institutional conditions

Within the governance domain, a lack of coordinated governance across sectors and limited cross-sector communication created significant blind spots in disease surveillance and response. Communities were largely excluded from conservation decision-making, a pattern that shaped both disease narratives and willingness to engage with formal disease control measures:

*“What's in the nature reserve belongs to the nature reserve; the community is not involved.”*

(COMM-02)

Rewilding decisions by conservation authorities and community stakeholders rarely considered diseases, but instead relied on assumptions by conservation authorities about pre-introduction health screening of wildlife. This overconfidence in biosecurity, combined with the absence of coordinated governance across private landowners, conservation authorities, park managers, and community members, further limited disease surveillance and response.

### 3.6 Scenario-based synthesis of disease transmission pathways

Based on our thematic analysis of interview results, we identified two conceptual scenarios illustrating stakeholder-perceived pathways linking rewilding, scavenger dynamics, livestock management, and disease risk.

#### 3.6.1 Scenario 1: rabies and anthrax mediated through scavenger dynamics and governance gaps

Stakeholders consistently identified scavengers, particularly vultures, jackals, and free-roaming dogs, as central actors linking wildlife, livestock, and human disease risk. In this scenario, rewilding and wildlife reinforcement increase herbivore biomass, thereby increasing carcass availability. Where vulture abundance and species richness are maintained, carcasses are rapidly removed, reducing environmental persistence and sporulation of *Bacillus anthracis*. Reduced vulture abundance and increased mammalian scavenging prolong carcass persistence, elevate exposure of dogs and jackals to infected tissues, and increase pathogen dose (Fig. 3).

**Fig. 3:**
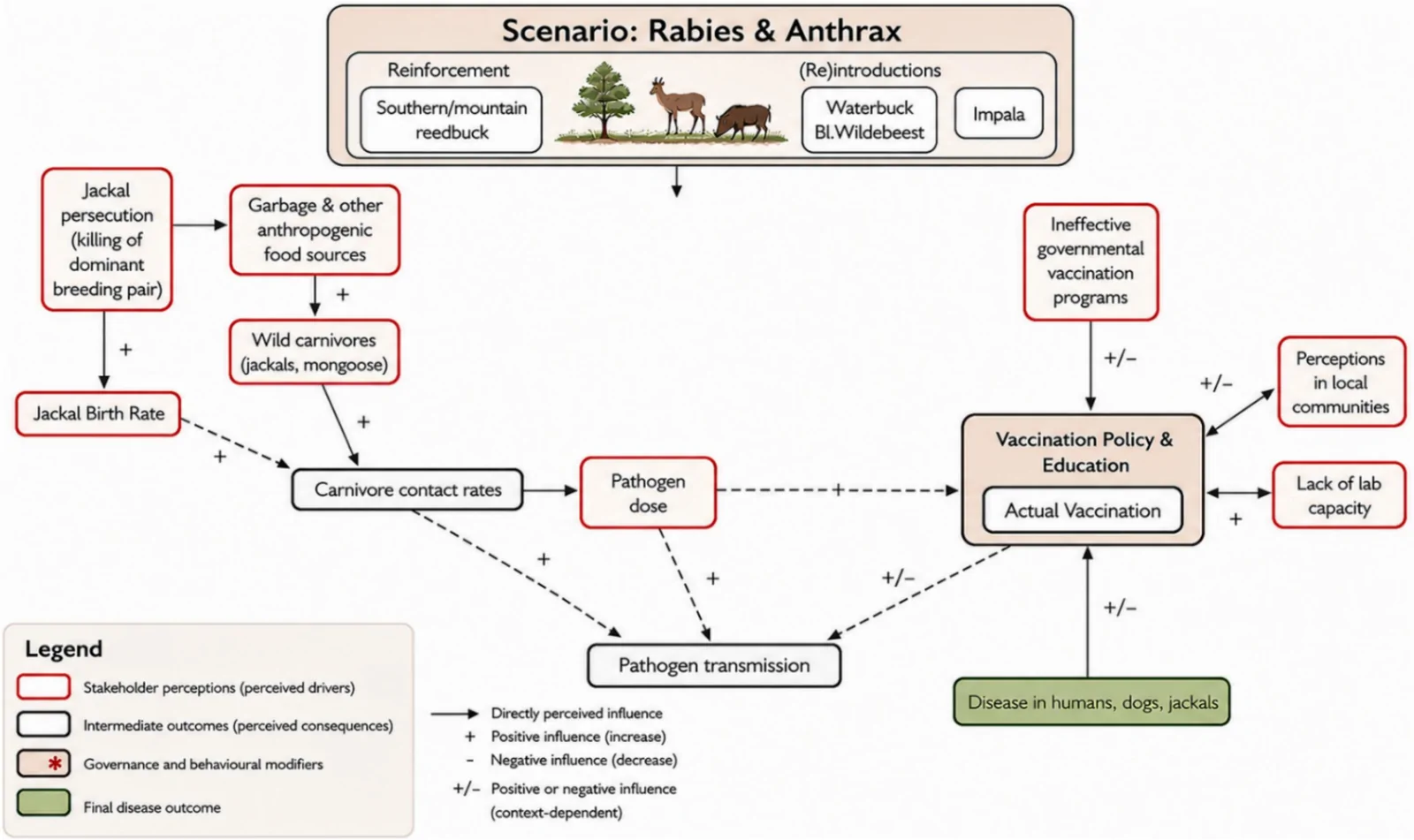
Stakeholder-derived conceptual pathways linking rewilding, scavenger dynamics, governance factors, and rabies/anthrax transmission risk in the Northern Drakensberg landscape, South Africa. Solid arrows represent directly perceived relationships among factors, while positive (+), negative (−), and context-dependent (+/−) symbols indicate the direction of perceived influence.

#### 3.6.2 Scenario 2: livestock-wildlife diseases under mixed farming and movement constraints

A second scenario focuses on directly transmitted diseases at the livestock-wildlife interface, such as brucellosis. Participants described communal grazing systems, mixed farming of cattle and small ruminants, and seasonal livestock movement as dominant features of the landscape. Rewilding and reinforcement of wild herbivore populations increase opportunities for spatial overlap with livestock, particularly where fencing is limited or absent. Stakeholders highlighted abortion events, disposal of fetal material in open environments, and contact between livestock and wildlife scavengers as key risk points. Poverty, transportation constraints, and lack of suitable vaccines for wildlife hinder effective disease control, while the absence of a coordinated disease management structure between private landowners, conservation authorities, park management, and surrounding communities further limits the ability to implement consistent surveillance and control measures across the landscape. These conditions create pathways for pathogen transmission through direct contact with aborted fetuses, shared grazing areas, and stress-induced pathogen shedding in pregnant animals during calving periods, when brucellosis transmission risk is highest. This scenario illustrates how disease risk in rewilding landscapes is shaped by the interplay of ecological processes, human behaviour, and institutional constraints rather than by rewilding actions alone (Fig. 4).

**Fig. 4:**
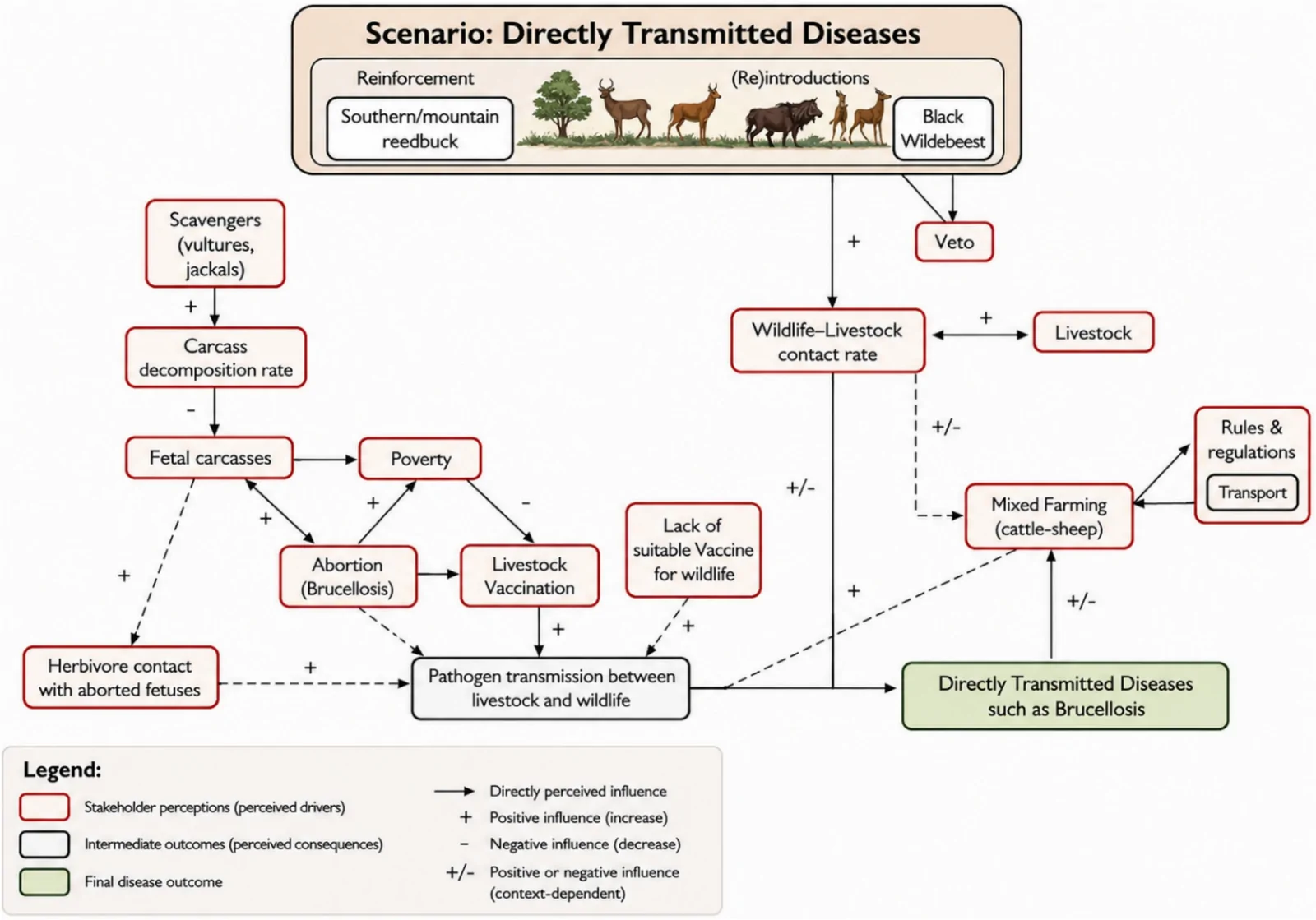
Stakeholder-derived conceptual pathways linking rewilding, livestock management practices, governance constraints, and directly transmitted disease risk at the livestock-wildlife interface in the Northern Drakensberg landscape, South Africa. Solid arrows represent directly perceived relationships among factors, while positive (+), negative (−), and context-dependent (+/−) symbols indicate the direction of perceived influence. Fetal carcasses are linked to poverty (other carcasses are not) because aborted fetuses were perceived as a direct loss of livestock offspring and income, which in turn was perceived to reduce vaccination of livestock.

In Fig. 4, fetal carcasses are linked to poverty, but other carcasses are not, because aborted fetal material was perceived to have a distinct set of consequences. Aborted fetuses are the most visible outcome of brucellosis in livestock and were therefore the carcass type most directly associated with this disease, in contrast to the carcasses of adult animals in Scenario 1, which were discussed mainly in relation to scavenger access and carcass removal.

Each aborted fetus represents a direct loss of offspring and future herd productivity. These losses were perceived to deepen poverty among households that depend on livestock, and poverty was in turn perceived to reduce the ability to pay for veterinary services and the vaccination of livestock, which sustains the cycle of abortion and pathogen transmission.

## 4 Discussion

Our study demonstrates that disease transmission risks in rewilding landscapes are shaped by the integration of ecological and social processes rather than by wildlife population dynamics alone [6]. In the Northern Drakensberg landscape of South Africa, a socio-ecological system characterized by heterogeneous land use, uneven access to resources, and historically embedded power imbalances between conservation authorities and local communities, rewilding shapes perceptions of disease transmission risk among different stakeholders. Stakeholder perceptions of disease risk were strongly shaped by livelihood practices, historical exclusion from conservation decision-making, and distrust of institutional narratives, collectively undermining disease surveillance and compliance with control measures. Furthermore, scavenger dynamics and wildlife-livestock contact emerged as key ecological transmission pathways, while a lack of coordinated governance and limited cross-sector coordination created critical surveillance gaps. Our insights align with broader One Health literature emphasizing interdisciplinary approaches to disease at human-animal-environment interfaces, though such integration remains underrepresented in practice [11], [31].

A central finding of this study is that rewilding is neither intrinsically protective nor inherently risky for infectious disease transmission. Instead, outcomes are highly contingent on how ecological changes intersect with social practices, land-use patterns, and institutional responses [6]. Emerging evidence suggests that well-designed trophic rewilding, particularly the restoration of apex predators, can, in some contexts, reduce the risks of zoonotic disease transmission through top-down regulation of reservoir host populations. However, such effects are highly context-dependent and contingent on social and land-use conditions [6]. In the Northern Drakensberg Nature Reserve, where communal grazing, human-mediated pressures on mesocarnivores, and limited veterinary access dominate, risks appear amplified rather than mitigated. Although conservation actors and park managers expressed confidence that pre-release screening minimizes risks from wildlife introductions, this overlooks post-release dynamics, including pathogen persistence, behavioural shifts, and altered contact rates [7].

Scavenger communities emerged as key mediators of disease risk in the ecological domain. Vultures were perceived as beneficial for rapid carcass removal, reducing the environmental persistence of pathogens such as *Bacillus anthracis*, consistent with evidence on obligate scavengers [32]. Conversely, mammalian scavengers such as jackals and free-roaming dogs were perceived to increase the risk of rabies transmission through repeated contact with infected carcasses. The persecution of scavengers and vulture population declines due to poisoning, as reported by the study participants, may prolong carcass persistence and shift reliance to facultative scavengers, with cascading effects on disease exposure pathways [33], [34]. This infers that functional restoration of scavenger communities, particularly vultures, should be treated as an integral component of disease risk management in rewilding landscapes, rather than solely as a conservation objective. Socio-economic and land-use factors further shaped disease transmission pathways. Communal grazing and limited fencing increased livestock-wildlife contact, practices that reflect adaptive responses to resource scarcity rather than mere tradition. Constraints on veterinary access limited vaccination coverage and disease reporting, reinforcing how economic marginalization shapes vulnerability to disease [10]. Seasonal livestock movement into highland areas during winter created additional opportunities for contact previously unrecognized as disease risks by local communities. Rewilding decisions in the Northern Drakensberg Nature Reserve prioritized habitat suitability, grazing pressure, and human safety, treating disease as a technical issue rather than a governance challenge requiring ongoing monitoring and cross-sectoral coordination [35].

Pronounced differences in disease awareness emerged among community members, conservation professionals, veterinarians, and healthcare workers, reflecting distinct institutional roles and lived experiences rather than mere knowledge gaps [36]. Community members' disease concerns were overshadowed by immediate livelihood pressures such as access to grazing and healthcare. Veterinarians focused primarily on livestock economics, while conservationists emphasized biodiversity. This fragmentation of understanding across stakeholder groups hinders a holistic approach to disease risk management at the human-livestock-wildlife interface. Mistrust rooted in historical exclusion from conservation decision-making further shaped disease narratives, with some community members viewing diseases such as rabies as externally imposed, perceptions that directly influence vaccination compliance and surveillance participation [37].

## 5 Limitations of the study

Despite gaining insights into stakeholder perceptions of disease transmission risks, we acknowledge some limitations. The sample size in our study was small (*n* = 24) and may not have captured the full diversity of stakeholder perspectives across the broader Drakensberg landscape; some stakeholder groups, such as healthcare workers and park managers, were represented by only two participants each. We considered the sample sufficient for a qualitative study of this kind because thematic saturation was reached during data collection, with no new substantive themes emerging in later interviews [38], but the findings remain context-specific and should not be read as representative of all stakeholders. It is also important to emphasize that the diseases discussed in this study, including rabies, anthrax, and brucellosis, are stakeholder-reported and perception-based rather than confirmed through epidemiological surveillance. The qualitative data do not constitute evidence of disease prevalence, incidence, or verified transmission pathways, and stakeholder perceptions may not capture all ecological and epidemiological details that quantitative surveillance would provide. Instead, they capture how disease risks are understood, narrated, and acted upon by different actors within a rewilding landscape. Likewise, the conceptual scenarios are interpretive syntheses of stakeholder narratives rather than quantitative predictions. The study is also limited to a single case study site, which constrains the generalizability of findings. Follow-up studies collecting quantitative data, for example on the use of veterinary and health services, disease prevalence and incidence, and carcass persistence and scavenger activity, and extending the work to other rewilding landscapes, would complement and test the patterns identified here.

## 6 Conclusion

Our study supports the One Health theory by empirically demonstrating that disease risk arises from socio-ecological and institutional interactions rather than through biological interfaces alone. The findings show that the fragmentation of governance responsibilities across conservation authorities, private landowners, and community stakeholders, along with stakeholder exclusion and livelihood precarity, are not merely contextual factors but constitutive elements of disease transmission pathways [10]. In doing so, this study responds to growing calls within the One Health literature for frameworks that move beyond biomedical approaches to engage seriously with power dynamics, political economy, and the lived realities of communities at the human-livestock-wildlife interface [10], [11], [39].

Furthermore, our study makes a strong case for embedding diverse stakeholder perspectives into rewilding governance as a necessary step toward putting One Health into practice and avoiding unintended health consequences. There is a need for disease governance that moves beyond sectoral boundaries and incorporates the social and institutional dimensions of disease risk [40]. Integrating disease considerations into rewilding as an ongoing, adaptive process rather than a one-time screening exercise is critical for anticipating post-release dynamics involving scavengers, livestock, and human practices [6]. Disease communication strategies should be tailored to socioeconomic contexts through inclusive community engagement, recognizing the functional role of scavenger restoration, especially vultures, as a disease mitigation strategy alongside its conservation goals [32]. One Health approaches should be operationalized at landscape scales through coordinated governance structures that meaningfully include community members [35]. Future research combining qualitative insights with quantitative and longitudinal data, such as health service use, disease prevalence and incidence, and carcass removal by scavengers, will be vital to ensure rewilding enhances biodiversity while safeguarding human and animal health.

## CRediT authorship contribution statement

**Samuel Akpan:** Writing – original draft, Methodology, Investigation, Formal analysis, Data curation, Conceptualization. **Helen Esser:** Writing – review & editing, Validation, Methodology, Investigation, Formal analysis, Conceptualization. **Rebekka Assink:** Writing – review & editing, Visualization, Methodology, Investigation, Data curation. **Laxmi Chaulagain:** Writing – review & editing, Visualization, Methodology, Investigation, Formal analysis, Data curation. **Frank van Langevelde:** Writing – review & editing, Supervision, Software, Resources, Project administration. **Percy Moyo:** Writing – review & editing, Methodology, Investigation, Formal analysis. **Gilian van Duijvendijk:** Writing – review & editing, Visualization, Validation, Supervision, Project administration. **Mbongiseni Shongwe:** Writing – review & editing, Visualization, Resources, Methodology, Investigation. **Kirsten Oliver:** Writing – review & editing, Validation, Resources, Methodology, Investigation. **Claudius van de Vijver:** Writing – review & editing, Supervision, Software, Resources, Project administration, Methodology. **Arash Ghoddousi:** Writing – review & editing, Visualization, Validation, Supervision, Software, Resources, Project administration, Methodology.

## Declaration of competing interest

The authors declare that they have no known competing financial interests or personal relationships that could have appeared to influence the work reported in this paper.

## Data Availability

Data will be made available on request.
