# Supplementary material for: Socio-ecological dynamics of disease transmission in rewilding landscapes: A case study of the Northern Drakensberg Nature Reserve, South Africa
Source: One Health. 2026 Nov 6;23:101653. doi: 10.1016/j.onehlt.2026.101653 (PMC13760704; doi:10.1016/j.onehlt.2026.101653)
Supplement: Supplementary file 1 [file mmc1.pdf]

Schedule

Date	Method Used	Category	People	Number
12/03	Interview	Ecologist	Sonja Kruger	1
13/03	Focus Group Discussion	Community People	Through Eco Monitor	5
13/03	Interview	National Park	Conservation Manager	1
		NGO	Kirsten	1
		Landowners	Vaun & Shaun	2
		Livestock Farmer		
14/03		Healthcare Professional		
14/03	Interview	Community head	Muzi	1
14/03	Interview	Agrovet Shop?	Manager?	1
14/03	Interview	Vet shop	Vet	1

Interview questions

Important:

- Introduce ourselves & explain why we are here/study objective
- Ethical considerations:
    - Interview is anonymous
    - Interview is voluntary
    - Feel free to decline answering any uncomfortable question
    - The interviewee can withdraw at any time during the interview

Livestock Farmers (1/2)

Goal: “we would like your perspective on diseases affecting your animals, and their interactions with wildlife”

Demography

1. What is your name? For how long have you been living here?
2. What is your occupation? Or are livestock your main source of income?
3. Where do you live? Or what is the name of this place?
4. How long have you lived here?
5. Gender (No need to ask)

Livestock and Diseases

1. How many livestock do you have?
2. Which type of livestock (and breed) do you have? E.g., Cattle, goat, sheep?
3. Since how many years are you involved in livestock rearing?
4. Have your animals been affected by any diseases? (*Probe further to know which diseases & symptoms, e.g. abortion, maybe show pictures)*
    1. *Foot & Mouth Disease*
    2. *Brucellosis*
    3. *East coast fever (theileriosis)*
    4. *Snot disease (Malignant Catarrhal Fever)*
    5. *tick-borne diseases (Babesiosis)*
    6. *Rabies in dogs*
5. How do you treat your livestock? *(probe further on what and how treatment is done)*

Rewilding Questions

1. What types of wild animals have you seen in this area? *Enlist.... Or guide with names.*
2. Have you seen any changes in the population of these or any of these wild animals?
3. Are there any new wildlife you have seen in recent years that were not here before? *(And vice versa)*
4. Which wild animals interact with your livestock? (*probe on the kind of interactions)*

5. How do you manage the interaction between livestock and wild animals in the area?

1. (*Reinforcement*) What do you think about increasing the population of the current wildlife in the DNR? Specifically, herbivores and vultures
2. Do you think this will ...
    a. affect your livestock? If so, how?
    b. influence disease risk in any way for either domestic animals or wildlife?

3. (*Reintroduction*) What do you think about reintroducing wildlife species in the DNR that used to occur here but are currently absent? Like buffalo.
4. Do you think this will ...
    a. affect your livestock? if so, how?
    b. influence disease risk in any way for either domestic animals or wildlife?

5. (*Introduction*) What do you think about introducing new wildlife species in the DNR that historically did not occur here?
6. Do you think this will
    a. affect your livestock? If so, how?
    b. influence disease risk in any way for either domestic animals or wildlife?

Vet (1)

Goal: "we would like your perspective on diseases affecting local animals, and their interactions with wildlife"

1. For how long have you been a vet?
2. Since when have you been a vet in this area?
3. Which animals do you mainly treat?
4. Which diseases occur in this area?
5. How many animals do you treat for these diseases each year?
6. Which vector borne diseases are occurring here?

7. What treatments do people do themselves?
8. Is dipping commonly done?
9. What is the status of acaricide resistance in this area?
10. How big of an issue is rabies?
11. How many dogs are vaccinated?

Explain rewilding
The next set of questions will be related to bringing back wild herbivores in this area and what you would think of that.

Rewilding questions

7. (*Reinforcement*) What do you think about introducing more individuals from wildlife species that already occur in the DNR?
8. Do you think this will influence disease risk in any way for either domestic animals or wildlife?

9. (*Reintroduction*) What do you think about reintroducing wildlife species in the DNR that used to occur here?
10. Do you think this will influence disease risk in any way for either domestic animals or wildlife?

11. (*Introduction*) What do you think about introducing new wildlife species in the DNR?
12. Do you think this will influence disease risk in any way for either domestic animals or wildlife?

Community Members (2)

Goal: “we would like your perspective on diseases affecting people and their animals, and their interactions with wildlife”

Demographic

1. What is your name? How many members are you in the family?
2. What is your occupation? Or main source of income?
3. Where do you live? Or What is the name of this place?
4. How long have you lived here?
5. Gender (No need to ask)

Explain rewilding
The next set of questions will be related to bringing back wild herbivores in this area and what you would think of that.

Rewilding questions

1. (*Reinforcement*) What do you think about introducing more individuals from wildlife species that already occur in the DNR?
2. Do you think this will influence disease risk in any way for either domestic animals or wildlife?

3. (*Reintroduction*) What do you think about reintroducing wildlife species in the DNR that used to occur here?
4. Do you think this will influence disease risk in any way for either domestic animals or wildlife?

5. (*Introduction*) What do you think about introducing new wildlife species in the DNR?
6. Do you think this will influence disease risk in any way for either domestic animals or wildlife?

Healthcare Professionals (1)

Goal: “we would like your perspective on diseases affecting people and their animals, and their interactions with wildlife”

1. How long have you been a healthcare professional?
2. Which diseases do you mainly treat?
3. Since when have you been a healthcare professional in this area?
4. Which zoonotic disease are a problem?
5. Do you see many rabies cases?

Explain rewilding
The next set of questions will be related to bringing back wild herbivores in this area and what you would think of that.

Rewilding questions

1. (*Reinforcement*) What do you think about introducing more individuals from wildlife species that already occur in the DNR?
2. Do you think this will influence zoonotic disease risk in any way?

3. (*Reintroduction*) What do you think about reintroducing wildlife species in the DNR that used to occur here?
4. Do you think this will influence zoonotic disease risk in any way?

5. (*Introduction*) What do you think about introducing new wildlife species in the DNR?
6. Do you think this will zoonotic influence disease risk in any way?

Conservation NGO (Kirsten)

Goal: “we would like your perspective on diseases affecting local animals, and their interactions with wildlife”

1. How long have you been working in this area?
2. Have you seen any changes in wildlife species occurrences or densities over the years? So species that came back or species that have disappeared?
3. Do you know of any diseases in domestic animals in the area?
4. Do you know of any diseases in wildlife in the area?

Explain rewilding
The next set of questions will be related to bringing back wild herbivores in this area and what you would think of that.

Rewilding questions

1. (*Reinforcement*) What do you think about introducing more individuals from wildlife species that already occur in the DNR?
2. Do you think this will influence disease risk in any way for either domestic animals or wildlife?
3. Do you know of a case where this influenced disease risk?

4. (*Reintroduction*) What do you think about reintroducing wildlife species in the DNR that used to occur here?
5. Do you think this will influence disease risk in any way for either domestic animals or wildlife?
6. Do you know of a case where this influenced disease risk?

7. (*Introduction*) What do you think about introducing new wildlife species in the DNR?
8. Do you think this will influence disease risk in any way for either domestic animals or wildlife?
9. Do you know of a case where this influenced disease risk?
10. Is disease risk something that is being considered by NGOs when considering introducing wildlife in an area?

Private land owners (2)

Goal: “we would like your perspective on diseases affecting local animals, and their interactions with wildlife”

1. What is your occupation or main source of income?
2. How long have you been living here?
3. Do you have any wildlife animals in your property?
4. Have you seen any changes in wildlife species occurrences or densities over the years? So species that came back or species that have disappeared?
5. Do you know of any diseases in wildlife in the area?
6. Do you have any livestock animals?
7. Do you know of any diseases in domestic animals in the area?

Explain rewilding
The next set of questions will be related to bringing back wild herbivores in this area and what you would think of that.

1. (*Reinforcement*) What do you think about introducing more individuals from wildlife species that already occur in the DNR?
2. Do you think this will influence disease risk in any way for either domestic animals or wildlife?

3. (*Reintroduction*) What do you think about reintroducing wildlife species in the DNR that used to occur here?
4. Do you think this will influence disease risk in any way for either domestic animals or wildlife?

5. (*Introduction*) What do you think about introducing new wildlife species in the DNR?
6. Do you think this will influence disease risk in any way for either domestic animals or wildlife?

Government Ecologist (1)

Goal: “we would like your perspective on diseases affecting wildlife and livestock, and the role of scavengers in modulating disease transmission”

Demography

1. How long have you worked in the conservation field? 28 years
2. Gender (female)

Rewilding questions

1. Which scavenger species are present in the Drakensberg conservation area?
    a. Bearded vultures foraging, historically breeding
    b. Cape vultures
    c. Raptors (facultative)
    d. Black-backed Jackals
    e. Brown hyena might squeeze in
2. How large is their population in the DCA (species + number)
3. What are the most important threats to scavengers in the DCA
4. What is their expected population trend → strong decline for vultures
5. What plans are there for reinforcing existing vulture populations in the DCA?
6. What plans are there for (re-)introducing vulture species in the DCA?
7. How will introducing new herbivore species affect vulture populations (new species expected?)
8. What are current perceptions of scavengers among local people (threats to livestock + feelings towards higher abundances).
    a. Do these perceptions differ among private landowners, livestock farmers, and other groups?
9. To what extent do vultures compete with other scavengers (jackals, dogs, ravens etc)?
    a. How important are dogs as scavengers in the DCA?
    b. How could that change if vulture populations go up?

Disease questions

10. What role do scavengers play in disease transmission in South Africa
    a. How does that differ between scavenger species
    b. Anthrax, bovine tb etc (only rabies, little education on other diseases)
11. What diseases are present in the Drakensberg region?
    a. Anthrax: Which species do you think will be most affected (Kudu? Impala? Eland? Any herbivore?)
    b. Are there seasonal dynamics?

Thinking about diseases! Will become part of the management plan but still working on it

Neo

Goal: “we would like to hear what you know about diseases in this area affecting local animals, and their interactions with wildlife”

1. How long have you been working in this area and what exactly did you do for your phd?
2. What kind of work do you do know?
3. Do you know of any changes in wildlife species occurrences or densities over the years? So species that came back or species that have disappeared?
4. Do you know of any diseases in domestic animals in the area?
5. Do you know of any diseases in wildlife in the area?
6. Do you know of any treatments that people use themselves?
7. Is dipping commonly done?
8. What is the status of acaricide resistance in this area?
9. How big of an issue is rabies?

Explain rewilding
The next set of questions will be related to bringing back wild herbivores in this area and what you and the community members would think of that.

Rewilding questions

11. (*Reinforcement*) What do you think about introducing more individuals from wildlife species that already occur in the DNR?
12. Do you think this will influence disease risk in any way for either domestic animals or wildlife?
13. What do you think the community’s perspective would be on this?

14. (*Reintroduction*) What do you think about reintroducing wildlife species in the DNR that used to occur here?
15. Do you think this will influence disease risk in any way for either domestic animals or wildlife?
16. What do you think the community’s perspective would be on this?

17. (*Introduction*) What do you think about introducing new wildlife species in the DNR?
18. Do you think this will influence disease risk in any way for either domestic animals or wildlife?
19. What do you think the community's perspective would be on this?

Vaughn

Goal: "we would like your perspective on diseases affecting local animals, and their interactions with wildlife"

1. Besides horses, did you have any other livestock animals in the past?
2. Have you had any diseases in your livestock?
3. Do or did you use preventative treatment for animals? Do most private landowners do so?
4. Do you know of any diseases in wildlife in the area?

5. Have you seen any changes in wildlife species occurrences or densities over the years? So species that came back or species that have disappeared?
6. When you want to introduce a new wildlife species, how do you decide which animal species? Is there a process or assessment procedure? Is there a list of species that used to occur here?
7. Do you think that buffalo could be introduced here?
8. You already mentioned the other landowner who did not want to introduce wildebeest because of the potential snotsiekte, do you have any concerns like that for specific diseases? Would you say that diseases are an important consideration when choosing to introduce animals?

Interview notes/transcription

Government Ecologist (Sonja 12-03)

Demography

1. How long have you worked in the conservation field?
   28 years
2. Gender (female)

Rewilding questions

1. Which scavenger species are present in the Drakensberg conservation area?
   Bearded vultures foraging, historically breeding
   Cape vultures
   Raptors (facultative)
   Black-backed Jackals
   Brown hyena might squeeze in
   Sometimes mongooses

   The bearded vultures stay year round, range up until 10 km from nesting site. Within 50 km there might be 2 other roosts.
   Jackal numbers are more linked to land use than to vulture numbers. They complement each other instead of competition. Cape vultures can open up carcasses for bearded vultures and jackals to feed on.
   Bearded vultures eat during the days, dogs and jackals come to feed at night. Cape vultures chase dogs away.
2. How large is their population in the DCA (species + number)
   See ppt → no breeding bearded vultures but the come in from other areas. A carcass might attract up to 50 vultures
3. What are the most important threats to scavengers in the DCA
   In this area, the main threat is poisoning.
4. What is their expected population trend?
   strong decline for vultures
   for Jackal, the population trend fluctuate depending on social dynamic and balances
5. What plans are there for reinforcing existing vulture populations in the DCA?
   No plans for Cape vultures, but to boost beard vultures
   If introductions are done, it is to boost populations, but only when threats are also taken care of. The Nature Reserve is a suitable area for these vulture species.
6. What plans are there for (re-)introducing vulture species in the DCA?

The only species that could be reintroduced is the Egyptian vulture, but there are currently no plans for that. But in southern part has more potential..

7. How will introducing new herbivore species affect vulture populations (new species expected?)
   Scavengers are not picky, but prefer probably smaller antelope species, so reintroductions are not expected to affect vulture species richness.
8. What are current perceptions of scavengers among local people (threats to livestock + feelings towards higher abundances).
   a. Do these perceptions differ among private landowners, livestock farmers, and other groups?
   Everyone hates jackals, because they are also predators and predate on livestock. Vulture perceptions are generally positive because they clean carcasses.

9. To what extent do vultures compete with other scavengers (jackals, dogs, ravens etc)?
   a. How important are dogs as scavengers in the DCA?
   b. How could that change if vulture populations go up?
   In communal areas jackal might eat antelopes, which is food for the communities, and they are also competing with dogs. Jackals and dogs might come in at night and eat the carcass before the vultures get to it during the day (note Helen: but jackals and dogs rarely completely finish a carcass)

Disease questions

10. What role do scavengers play in disease transmission in South Africa
    a. How does that differ between scavenger species
    b. Anthrax, bovine tb etc (only rabies, little education on other diseases)
    The role of vultures is debated; they could either control disease by cleaning up carcasses but also spread disease via blood etc that sticks to legs and head. But generally, scavengers limit the spread of disease.
    In terms of disease transmission, the main interactions are amongst carnivores (dogs, jackals, mongoose). No aggressive interactions among dogs and jackals, they avoid each other. Lots of interaction between livestock and game, so we need to be careful what we stock where. We only introduce species that are or were originally occurring in the area. If we were to introduce predators, e.g. lions, then we have to discuss this with the land owners because predators might break out and kill livestock. As for the communities around the nature area: they are not consulted, the private land owners who work with local livestock farmers would have to discuss with them.
11. What diseases are present in the Drakensberg region?
    a. Anthrax: Which species do you think will be most affected (Kudu? Impala? Eland? Any herbivore?)
    b. Are there seasonal dynamics?

Snotsiekte is a real problem in the area, carried by wildebeest and cattle. There have been a few anthrax and FMD scares in the wider area around Drakensberg, but it never really happened here. No problem with ticks here or brucellosis. The area is lucky with low temperatures. There is also no bovine tuberculosis that she has heard of, only in the warmer areas outside the Drakensberg region. Rabies does occur here, in dogs, vervet monkeys and jackals. There are vaccination programs and there is education on rabies, so people should know. Besides the low temperature, the big size of the park helps with preventing disease outbreaks (=lower population density). Diseases will become part of the management plan but still working on it.

Community members 13-03

They mostly work with plants and not animals

Q: What is your occupation?
A: We take care of nature by clearing alien plants. These plants differ in many ways, such that they outcompete native plants.

Q: Do you think that has an impact on livestock?
A: Yes, because by outcompeting native vegetation, they reduce the amount of pasture available. So, we clear them using both mechanical and chemical methods

Q: Do these methods have any effect on the native vegetation?
A: No, because we are trained on how to use the pesticides and to precisely remove alien vegetation.

Q: Do you know about any diseases affecting livestock in the area?

A: Yes, we know of foot and mouth disease (Present in the area).

Q: Is your livestock allowed to graze within the nature reserve?

A: No, not at all. The only allowed cows are those owned by white people (Private farmers), but ours are not allowed at all. It happens that if they find ours inside, you might have to pay to get them released because they are found where they should not be.

Q: Are there any interactions between wildlife and livestock

A: Yes. It's only with the cows owned by private farmers.

Q: How abundant are wild animals in the DNR?
A: There is high abundance but low diversity

Q: Which animals are present in the DNR?

A: Baboons, Eland, Warthogs, Porcupines

Q: Do you know about any diseases affecting wildlife in this area?
A: No, unfortunately, we don't work with animals. Only vegetation.

Q: What are your thoughts on new animals being introduced in the DNR?
A: I think it is a good idea, No carnivores

Q: Do you think the introduction of wild animals in the DNR will influence disease risk in any way for either domestic animals or wildlife?
A: (Collectively) We have no idea about diseases associated with wildlife.
: (Responded 1) In my opinion, I am not informed about diseases, but I think if an animal is moved from one place to a new place, it will not be used to the type of pasture available and so will suffer. But it also happens that it quickly adapts to the new habitat. So, I think it is important to first test if the environment is compatible with the animals to be introduced.

Q: How do you think rewilding will affect the environment (food)?

A: The grass here is diverse. It is not the same, we have *Sporobolus africanus*, *Aristida junciformis*, etc. The place is huge, so it will depend on where most of the animas are concentrated.

Q: Do you own livestock?
A: Yes, we do

Q: What livestock do you own?
A: Cows, goats, chickens, horses, dogs (companion)

Q: How do you herd your livestock?

A: There are herders available to herd multiple cows for hire.

Q: How do you treat sick livestock?

A: There is a wildlife vet available at Bergville.

Q: How does that work?
A: We note all the symptoms and describe them to the vet, and they tell us what is wrong.

Q: In the community, how do you treat/ prevent diseases in livestock cows?

A: Each village has its own dip. When we are supposed to dip, there will be someone to notify the village, then each herder will gather all the cows they herd to the dip.

Q: What role does this communal herding have on disease transfer between cows?

A: (Respondent 1) There might be a problem where a sick cow from one household transfers a disease to a cow from a different household. However, this does not occur in the village.

(Respondent 2) If you own livestock, you have two things. You have a camp and a kraal. If your cow gets sick and you don't know the reason, you conduct visual assessments such as injuries, before consulting the vet. But if it stops eating, always lay down, you isolate the cow. Because as you can see, this is a hilly place, you wouldn't want your cow to go up and down the hills in its condition. So you only allow it to graze in the camp so you can closely monitor it and gather all the necessary information to provide to the vet, until your cow is in good health.

Q: Are rabies present in the area?

A: Yes

Q: What animals do they affect?
A: Mostly dogs

Q: Where do you suspect the rabies come from?
A: (Responent 1) I have no idea, but In my opinion, I think it's a "dogs" disease that occurs. For example, you know how children are supposed to be vaccinated for certain diseases. So do dogs. But to say there is an origin, I wouldn't know

(Respondent 2) In my opinion, rabies, like all diseases present in the world, are a conspiracy. Developed countries manufacture these diseases for profit and economic development. Because if they introduce diseases, such as rabies, they also produce vaccines for themselves, and then sell them for profit to developing countries like us.

(Respondent 3) In my opinion, rabies comes from poor sanitation around dogs. We don't typically wash our dogs like white people do, we don't. Our dogs are also free to roam and eat whatever is available, which is what I think contributes to them getting rabies.

Q: Are there any wildlife-dog interactions within the nature reserve (Dogs from the community entering the reserve)?

A: Yes.

Q: How does that happen?

A: Since there are guards in the DNR, there are people who want to hunt illegally, so they train their dogs to precisely hunt their target. Dogs do not just suddenly come within the reserve. If they are here, there are with someone.

Q: Do you think rewilding will increase these wildlife-dog interactions?

A: I am not sure about illegally, but legally, there is an agreement between the nature reserve and the chief. Where we have annual hunts for the community and the chief, where a certain number of animals are hunted.

Q: (Again) What are your opinions on rewilding?

A: We think it is a alight. It depends on what kind of animals they introduce.

Q: What other general problems do you wish to talk about?

A: (Respondent 1) From my side, my brother I work with invasive plant clearing. Everything else, such as animals, we know here and there but don't have a lot of information.

(Respondent 2) From my side, I would like to see change regarding pasture available for livestock in communities. Because there are times where there is crop farming, which

reduces the amount of space available for livestock. So livestock ends up overgrazing in smaller, steep areas where they end up trampling and causing erosion.

(Respondent 3) Also regarding the amount of pasture available for livestock, If it were up to me, I would introduce fencing around green pasture, allow it to recover, and introduce rotational grazing where fences act as physical barriers to prevent forced grazing of recovering sites.

Q: (Again) What are your thoughts on rewilding?

A: I think it is a good thing because it ensures that our grandchildren will be able to see those animals.

Q: Would the broader community agree with that?

A: The community has no opinion. What's in the nature reserve belongs to the nature reserve; the community is not involved. As you can see, there are tours. All things done here (Nature reserve) are done for other people such as tourists. But as long as its not carnivores, the broader community would not have a problem with it.

Q: (Again) Are there any interactions between livestock and wildlife?

A: (Respondent 1) In my opinion, yes. Ther are some negative interactions. It happens that, for example, a dog bites a cow and has rabies, which creates problems for the cow.

(Respondent 2) There are also reedbucks which commonly come to the community, resulting in people hunting it with dogs.

(Respondent 3) There are also interactions between Eland and cows that result in interbreeding.

Royal Natal Park Manager 13-3

1. People: Benedict Nene (Male) from Royal Natal National reserve
2. How long have you been working in this area?

   7 years

3. Have you seen any changes in wildlife species occurrences or densities over the years? So species that came back or species that have disappeared?
   Eland populations have increased, currently more than 300 individuals occur in the park, almost 500. Elands are the dominant wildlife species. Other species are for example baboon, bushbuck, klipspringer, mountain reedbuck, wild pigs. Only the eland are increasing, other species are staying consistent. This is due to the protection of the park and less illegal activity. There are 3 zones, the outer zone does not have eland.
   Predators that occur here are mainly vultures and jackal.
4. Do you know of any diseases in wildlife in the area?
   FMD in buffalo in other areas north of KZN. Also rabies outbreaks, but not specifically here. He did not hear about any disease occurrence or outbreaks in the national park. Most animals die of old age or other natural causes, but dead animals are reported and samples are taken to be tested.

1. (*Reinforcement*) What do you think about introducing more individuals from wildlife species that already occur in the DNR?
   That is not necessary, but decisions are being made by local park ecologists after assessing the area. There is no fence in the upper escarpment.
2. Do you think this will influence disease risk in any way for wildlife?
   Introducing animals is no issue because animals can roam far. Because of this large range of Drakensberg, there is also no culling in the park.

3. (*Reintroduction*) What do you think about reintroducing wildlife species in the DNR that used to occur here?
   There are no plans to reintroduce animals that used to occur here in the past.
   It is important to only introduce animals that naturally occur here, to ensure their survival.
4. Do you think this will influence disease risk in any way for wildlife?
   The main risk for introducing animals is whether this area provides suitable habitat, diseases are not considered a risk. He does not see any risk associated with introducing animals/species regarding disease because they will be checked beforehand.

5. (*Introduction*) What do you think about introducing new wildlife species in the DNR?
   The main concern again is whether the animals are appropriate/suitable for the area, not disease-related.
6. Do you think this will influence disease risk in any way for wildlife?
   Not necessarily because only healthy animals will be introduced.

Info not related to questions:
He is not aware of any leopards in the park, he has never seen one, but it has been sighted nearby.
There are no zebra in the park.
Now, there is also lots of wild pig and jackal. The jackals are considered a problem and are not conserved. They kill bushbuck and are considered a problem.
Cattle/livestock are not allowed in the park. Fences are sometimes cut so livestock can go in, but this is being checked daily. The park presses charges against farmers who are caught.
There is an agreement between park and local communities for traditional hunting (Amazizi tribe), in the part of the world heritage site, not the park.
The tick problem is only mild, all animals have ticks, but he does not know of any cases where this was an issue or gave diseases.

Neo (PhD in communities) 14-3

1. How long have you been working in this area and what exactly did you do for your PhD?
   She started her PhD in 2019 on the quality of soils and small scale farming. It was initially very soil-focused, but that did not turn out to be the main issue for farmers, so she changed to a farming systems approach. Access to water is the main issue. People get water from running streams but due to climate change they have changed to seasonal streams. The terrain is also not easy to navigate to get water. So know they only farm a smaller portion of the land that they own. Another factor is labor, many people are old and can only do what their body allows them to do because they can not hire other people.

Livestock is not intensively managed but are just allowed to roam freely. Only in the night they come back to the kraal. Only a few families can afford herdsman to guard the livestock. But even then the cattle will be herded around the community, which is why crop production is so low. There is no fencing around the fields. But now arrangements are made so the livestock can eat remains after harvest.

2. Do you know of any diseases in domestic animals in the area?

   Every know and then there is an outbreak, not big, not really anything she knows of in this area specifically. FMD has been in KZN, not in this particular area. People make traditional remedies. Because of money they cannot go to a vet, but they also rely on indigenous knowledge because they are used to do it. The communities also work with the NGO Mahlathini which is very instrumental with helping them get to resources on time when there is an outbreak. They advise on what to use during a disease, and help with transport from the town and back to get medicine. The focus of the NGO is more on how to shift the focus to more conservation-focused agriculture. But even know and then they do elements of livestock production. Recently, another organization (meat naturally) started working on education farmers on rotational grazing as benefit for the soil and livestock, and the help to organize auctions for example.

   FMD is the main disease, she has not heard of other disease outbreaks in the area/surroudings.

3. Do you know of any diseases in wildlife in the area?

   No

4. Do you know of any treatments that people use themselves?

   Traditional remedies, also preventative. They put aloe vera in the drinking water of chickens as a preventative measure for health.

5. Is dipping commonly done?

   There was a facility, but is not very functional at the moment. That was used as a preventative measure. There is lots of ticks in the area, but they have learned how to manage them well. Besides dipping, spraying is also commonly done.

Dipping is not being done just against ticks but as a preventative measure against all kinds of diseases. When there is a threat in the area, then everyone gets together to organize dipping.

6. What is the status of acaricide resistance in this area?

   This is not really an issue in this area, the treatments work well here.

7. How big of an issue is rabies?

   Rabies cases are incidental, it is not a big issue. She mentioned a possible case she heard of when she was home during covid, but she does not know if this was true and where the dog got rabies.

Rewilding questions

1. (*Reinforcement*) What do you think about introducing more individuals from wildlife species that already occur in the DNR?
   Increasing the competition for grazing if they are introduced in the non-fenced areas. In the winter months, the livestock is taken up into the mountains for grazing and then there could be competition for the grass in the non-fenced areas. The winter months are therefore the months these animals interact, so diseases that have an outbreak then could be a risk. From a farmer perspective, however, the competition is likely a larger issue than potential disease transmission.
2. Do you think this will influence disease risk in any way for either domestic animals or wildlife?
   If you would introduce them in the park, this would not give any problems because the animals would be fenced in. So they would be separate from communities. But other parts of the mountain range are not as protected, so if the animals would be there, there could be issues. The problems would be more for the communities in Lesotho then here, because they might want to trade in new animals and therefore steal them before the disease has a chance to spread here.
   Ezemvelo have started to allow communities to enter the park for harvesting medicinal plants (and traditional hunting), and they also want to dedicate a portion of the park for grazing in the winter months. Because otherwise it happens uncontrolled when the grass runs out outside the fenced areas during winter and they want to graze their cattle on better quality grounds.
3. What do you think the community's perspective would be on this?

Theft is the largest issue for livestock farmers, not diseases. Animals get stolen if you don't have a herdsman by people from Lesotho.

4. (*Reintroduction*) What do you think about reintroducing wildlife species in the DNR that used to occur here?
   She is more curious about the wildlife itself, whether they are adapted to survive here with all the changes that occurred in the surroundings.
5. Do you think this will influence disease risk in any way for either domestic animals or wildlife?
   Similar to previous question
6. What do you think the community's perspective would be on this?
   Their day-to-day live is not really affected by this, so they will not really care, unless it is carnivores that are being introduced. They can eat livestock so affect day-to-day live. Even with fenced-in carnivores they would not be happy, since then the fence becomes a 'hard border': they cannot just cut the fence for cattle grazing in the park.

7. (*Introduction*) What do you think about introducing new wildlife species in the DNR?
   If it improves my life, go ahead, if not, I don't care as long as it is not carnivores.
8. Do you think this will influence disease risk in any way for either domestic animals or wildlife?
   Similar to previous question
9. What do you think the community's perspective would be on this?
   Similar to previous question

Muzi Community head – (14-03)

Lived here since 1996 (28 years)

1. Do you know about any livestock diseases?
2. Interaction of livestock diseases with human?
   There is not so much except for ticks. However, I don't know much about the diseases. There is few problems of Rabies from dogs. The hunting dogs don't really go into the communities.

3. What wild animals have you seen in the area?

a. Eeland

b. Rabbits
c. Monkeys
d. Ginny fowls
e. Bucks

4. Interaction about wildlife and livestock diseases?
   There is not so much of problem. Priority for disease is less here.
5. All sorts of rewilding perspective
   Yes, positive. It will be good to see more wild animals and for tourism. The only concern is the safety for human.
6. How nature and conservation is perceived in the community?
   Conservation is not in the priority. Community is not motivated about conservation of wildlife. The high school students are taught who follows natural science. In the schools, this is not focused.
7. How do community perceive wildlife?
   Community people are quite separated from the wildlife. However, they hunt and are interested in hunting.
8. fg

Not related questions

9. How do you identify rabies?
   We just assume looking at the dogs' symptoms or when it dies. He doesn't know about any inspection about the rabies.
10. People use dogs for hunting. Hunting is common in the area for tradition, it is a traditional game (like fishing), and also bush meat. Some people live on bush meat.
11. Community people and conservationist relationship
    Community people used to be a part in the past, but now involvement of community people in decision regarding conservation (or rewilding) is done alone by national parks or other people.
12. HIV was a problem in the past which is mitigated now with the USAID fund. Now in problem due to US withdrawing the aid.

Animal food supplier (14-03)

Q: From your experience here, how long have you been here?

A: About a year

Q: In terms of animal diseases, what kind of animal diseases are around Bergville?

A: It varies, but you do get quite a lot of diseases that the guys will come and report, then you have medications like antibiotics that is sold to customers.

Q: Do you know if ticks are a problem in this area?

A: Yes, they are, because I have seen them several times. People do come to buy dip, but there is not case of acaricide resistance.

Q: Have you heard about acaricide resistance?

A: Most of the customers that buy from us don't come back to give a report to say that the medication is not responding. They will buy, and you will only see them once the medication is finished.

Q: From your view, are they mostly pet owners or livestock owners?

A: We get both

Q: Do you sell any drugs for wildlife?

A: No, not here, only our main branch. We only sell for livestock and pets.

Q: Do you have foot and mouth disease in the area?

A: More especially with cattle, yes.

Q: How about rabies?

A: Yes, with the dogs especially. But it is not a major problem because the government has vaccination programs.

Veterinary Shop (Simon) – (14-03)

1. What livestock diseases areprevalent in the areas?
    a. Red Water (Babesiosis)
    b. Blue Tongue

   c. Black quarter
   d. Pneumonia
   e. Horse sickness (issue)
   f. StIff sickness?? (walk slowly)
   g. Scape worm mostly by sutu people??
   h. Lumpy Skin disease (issue increasing)
   i. Ticks
2. How often are ticks treated?
   By dipping. Seasonal variability, in rainy months once per 2-3 weeks and in non-rainy days once per 3 months.
3. Interaction of wildlife and livestock in the area in terms of diseases?
   He doesnot think so.
4. gh

Simon (Vet assistant?) – 13/03

Q: How long have you been working in the area?

A: Over 30 years (30-something years)

Q: In these 30-something years, what are the diseases you have experienced, mainly in the Drakensberg area?

A: Bovine Pneumonia, red water (bovine babesiosis), Stiff sickness (Bovine Ephemeral Fever (BEF) – Starting now), Lumpy skin disease, Blackquarter, Gallsickness (anaplasmosis), and African horse sickness.

Q: All these diseases, do they affect wild animals too?

A: No, there are no wild animals on this side

Q: Do people come from Drakensberg to buy medication?

A: They mostly buy Ivomec for deworming and acaricides.

Q: Do people from the Amazizini community come and buy medication here?

A: Yes, they do

Q: What kind of medication do they buy?

A: Medication for red water (bovine babesiosis) and Gallsickness. Not so much lumpy skin because people are learning to vaccinate.

Q: Do you think that there're is a problem in places like Amazizi, that wild animals can be giving some diseases to livestock?

A: (Directed us to Tukela vet)

Q: Have you ever recorded if there were diseases that pass from wildlife to livestock?

A: No, only in places where people have a forest.

Q: What about rabies?

A: Rabies no. Not here.

A (Randomly): In the mountains, at Amazizini they have Blesbok and Eland.

Hloniphile (Operational manager - Clinic)

Q: How long have you been practicing here?

A: 3 years

Q: In your view, what are the common diseases that you have mainly encountered here?

A: The most prevalent one is Tubercolosis, I simply think because it is a droplet infection

Q: What about in children? (Pointed out the common diseases in the past financial year posted in the bulletin board)

A: For the past year, we have not had pneumonia, but malnutrition is still there, though not prevalent because in the whole financial year, there was 1. But it will decrease because of the outreach services provided by our nutrition adviser.

For youth, we have a lot of STDs as well as MUS (Muscle disorders), and HIV/AIDS.

Q: Do you know about zoonotic diseases? Like rabies

A: Usually, for us in rabies management we have a protocol on how to treat people that have been bitten by dogs and cats. Ever since I came here, we have never had a positive case of rabies.

We have categories do deal with bites

We treat Category 1 (Bruises, no cuts, no sores, no sepsis) and category 2 using antirabies. Category 3 and 4 we refer to hospital to also get the immunoglobulin.

The treatment is within 24 hours of the incident

Q:What about water communicable issues?

A: We don’t have issues in the clinic.

Q: For the community?

A: For the community, nothing has been done, but we have never had cases of E. coli. Although some people come complaining of diarrhea diseases, that’s not because of water because we have never had a positive for E. coli.

Q: What does it feel like working in a facility near a nature conservation area?

A: Unfortunately, you must have money just to see the “beautiful” nature like animals.

Q: Do you think the environment (nature reserve) is positively or negatively affecting the disease problems that you see?

A: No, because even if you can have diseases that are caused by ingesting meat from a slaughtered cow. Those cattle are from the community and not from the reserve.

Also, the nature reserve is creating job opportunities for people in the area.

Vaughn 15-03

Goal: "we would like your perspective on diseases affecting local animals, and their interactions with wildlife"

1. Besides horses, did you have any other livestock animals in the past?

   Cattle

2. Have you had any diseases in your livestock?

   Invasive plant in the area is a problem: Lantana, poisonous, light sensitivity, animals can die

   Gall sickness and red water (ticks are a problem; battled with both in the past), three day stiffness, pink eye (Kerato-conjunctivitis), African horse sickness, heart water, brucellosis, btb. These disease all occur in this particular area. Brucellosis and tb over SA. Cross breeds between zebu and Bos taurus cattle to make them more resistant to ticks and diseases.

3. Do or did you use preventative treatment for animals? Do most private landowners do so?

   Cattle farmers gall sickness tetracycline treatment. Berenyl for red water, but you have to inject soon. Preventative treatment not so much, but slowly create immunity and not dip so often. Dips they used become more bird friendly. Acaricide resistance is not a problem in this area. In winter they dip less because less ticks. Except for regularly dipping against brown ear tick in winter.

   Resistance to antihelmintics is becoming more problematic in sheep farms. Wire worms deplete sheep of 8 l a day and sheep dies form anemia. Wildlife are not affected by them neither are horses.

4. Do you know of any diseases in wildlife in the area?

5. Have you seen any changes in wildlife species occurrences or densities over the years? So species that came back or species that have disappeared?

6. When you want to introduce a new wildlife species, how do you decide which animal species? Is there a process or assessment procedure? Is there a list of species that used to occur here?

   Waterbuck occurred here. Both southern and mountain reedbuck occur here now. Work closely together with KZN wildlife regarding (re)introductions, trying to get together a parcel of breed stock from Ezemvelo wildlife park to introduce animals from.

   Zebra numbers have gone up in this area (from 16 to 42). Horses used to be afraid of zebra but not anymore
7. Do you think that buffalo could be introduced here?

   Buffalo could do very well here in the area, but they could kill you/lead to human-wildlife conflict. So not on the list of potential introduction species (but who knows in the future).
8. You already mentioned the other landowner who did not want to introduce wildebeest because of the potential snotsiekte, do you have any concerns like that for specific diseases? Would you say that diseases are an important consideration when choosing to introduce animals?

   No wildebeest introductions because of snotsiekte, even though they could be potentially clean.

   From west to east its safe to move cattle in the country because of tick-borne disease, cattle here are more immune to ticks. Breeds are mixed with zebu and more resistant to red water, horse sickness and cowdriosis (heart water).

   Herds are checked for brucellosis across the country.
9. Do you allow cattle grazing on your land?

   Fire is the main management and is done by rotation on his property. No introduction of other cattle from farmers on his property, to prevent erosion etc. It used to be overgrazed, now grazing pressure is reduced. Now recovered. So, he doesn't want cattle in the farm. He might want to introduce a small herd, but very

controlled, but unlikely, as alternative form of income. Using a whip with cattle might chase game away and cattle trample soil and impact washoff, so that is unwanted.

10. Are there places where livestock and wildlife can interact, for example through drinking from the same water source?

There are 6 wells where cattle and livestock both drink, 18 that are zoned for development for making small dams. Atm not enough for drinking spots, he wants to stay elands as long as possible on the farm for tourism. He did not think it is a problem for transmission between wildlife and his horses.

Veterinarian 16-03

Interview veterinarian livestock owner

- Has lived in the area all his life
- Was convinced to join to NDNR
- Ticks are a big problem; prevalence of Heartwater, Redwater and Gall sickness is high. If you bring in cattle from outside, they will suffer because they don't have resistance. When he dips his cattle, he leaves a few ticks to induce resistance in his cattle towards ticks
- 4 methods of dipping: 1) letting cattle swim through a pool of acaricide; difficult to control the concentration, it goes down with every dip, 2) spraying by hand, 3) spraying…? 4) one point of dipping on the head, drips down, but never reaches the belly
- Acaricide resistance is a big problem, particularly in the surrounding communities
- Rabies: there were very good free (!) vaccination programs but the Faculty of Veterinary Medicine has fallen apart. So lack of vaccination has nothing to do with poverty in local communities, as veterinary teams would go out and vaccinate for free. Rabies is purely a dog problem. Likewise, whenever there is an outbreak of food and mouth disease, in the past they would block roads and impose transport restrictions to contain outbreaks but that's all gone. It's an atrocity.
- He is fine with introducing wild herbivores, but wildebeest are an absolute no-go because of snotziekte. A disease of close contact (note Helen: not true, direct contact does not spread the disease but eating grass in areas contaminated with saliva of wildebeest). All wildebeest are latently infected, so introducing 'clean' wildebeest is not a possibility.
- Bovine tb and brucellosis occur all across the country, buffalo important host. But foot and mouth disease is a far more important disease.

- Kudu zitten in lager gelegen delen en op deel van zijn reserve, net als eland. Eland dragen een specifiek soort Amblyomma teek (bonte teek) die besmet zijn met Heartwater en sinds de eland zijn toegenomen zijn de problemen met teken ook toegenomen. Helen: zijn eland geïntroduceerd of waren ze altijd al hier? Ze waren volgens mij altijd al hier maar in lage dichtheden. Volgens mij zijn er dieren ontsnapt vanuit Spioenkop game reserve en komt daar ook een deel van de ziektes vandaan.

Summary of Interviews

1. Which wild animals are present in the Drakensberg conservation area?
    A. Scavengers
        a. *Bearded vultures*
        b. *Cape Vultures*
        c. *Raptors*
        d. *Black-backed Jackals*
        e. *Brown Hyena*
        f. *Mongoose*
    B. Herbivores
        a. *Eland*
        b. *Baboon (omni)*
        c. *Bush buck*
        d. *Klipspringer*
        e. *Mountain reedbuck*
        f. *Wild pigs*
    C. Carnivores
        a. *Jackal*

2. Wildlife diseases in the DCA?
    a. *FMD*
    b. *Rabies (occasionally, not significant)*
    c. *Snotziekte*
3. Livestock diseases in DCA nearby areas
    a. FMD
    b. Rabies
    c. Red Water (Babesiosis)
    d. Blue Tongue
    e. Black quarter
    f. Pneumonia
    g. Horse sickness (issue)
    h. Stiff sickness?? (walk slowly)
    i. Scape worm mostly by sutu people??
    j. Lumpy Skin disease (issue increasing)
    k. Ticks

4. Reinforcement plan
    a. *Community People: No carnivores (predators)*
    b. *Ecologist: boost beard vulture population*
    c. *Conservationist: Not necessary*
    d. *Landowners:*

    e. *Livestock owner:*
    f. *Vet:*
    g. *Healthcare Professionals:*

5. Reinforcement impact on Diseases
    a. *Community People: NA*
    b. *Ecologist: boost beard vulture population*
    c. *Conservationist: Not necessary*
    d. *Landowners:*
    e. *Livestock owner:*
    f. *Vet:*
    g. *Healthcare Professionals:*
6. Reintroduction plan
    a. *Community People: No carnivores*
    b. *Ecologist: possibly Egyptian vulture, no plan currently*
    c. *Conservationist: No plan, maybe Leopard?*
    d. *Landowners:*
    e. *Livestock owner:*
    f. *Vet:*
    g. *Healthcare Professionals:*

7. Reintroduction impact on Diseases
    a. *Community People: NA*
    b. *Ecologist:*
    c. *Conservationist: No risk*
    d. *Landowners:*
    e. *Livestock owner:*
    f. *Vet:*
    g. *Healthcare Professionals:*

8. Introduction Plan
    a. *Community People: good idea,*
    b. *Ecologist: No plan*
    c. *Conservationist: No plan*
    d. *Landowners:*
    e. *Livestock owner:*
    f. *Vet:*
    g. *Healthcare Professionals:*

9. Introduction impact on Diseases
    a. *Community People: No idea*
    b. *Ecologist: NA*
    c. *Conservationist: NA*
    d. *Landowners:*
    e. *Livestock owner:*

f. *Vet:*
g. *Healthcare Professionals:*

7.
8.
9.

TABLE

**Stakeholders**	**Response to Rewilding**	**Why**	**Disease**	**Other concerns**
Community and Community Head	Positive	Visual and Tourism	No idea	• Feasible environment<br>• Protection of livestock (Fencing)<br>• No Predators<br>• Safety for human
Government (Vultures)	No plans			• Feasible environment<br>• Mitigate the threats<br>• Occur in the past
NGO	Not necessary	Plenty of space	No risk	• Feasible environment<br>• Occur in the past
University	Positive		Potential risk for livestock	• Competition for grass for livestock<br>• No Carnivores
Private Land Owner			Snotziekte	• No Wildebeest
Vet	Negative		Snotziekte	• Hunting,<br>• Not enough Protection (fencing)
Human Health Professional	NA	NA	NA	NA
Game Reserve	Positive	Economy	Not concerned	• Occur in the past

Components to include in framework

FMD incidence
Rabies incidence
Brucellosis incidence?
Snotsiekte incidence?
Babesiosis incidence?
Lumpy skin disease incidence?
More diseases?

Tick abundance
Treatments? Dipping? Traditional remedies?
Temperature (Lower disease incidence (FMD) due to lower temp?)
Seasonality? (Increased grazing in winter in park = increased contact?)

Host list

Anaplasmosis (gall sickness)

Wildlife hosts for A. marginale

- African buffalo: significant reservoirs
- Nyala: known reservoir, also for other tick-borne pathogens (Ehrlichia)
- Impala
- Kudu

Babesiosis (red water disease)

Wildlife hosts for B. bovis, B. bigemina

- African buffalo
- Water buffalo
- Antelope species uncertain

Brucellocis

Wildlife hosts for B. abortus, B. melitensis

- African buffalo (B. abortus, strain affecting cattle)
- Impala (B. melitensis, strain affecting sheep and goats)
- Kudu (B. melitensis)

- Other antelope species, but is vague

FMD (check if can be grouped with brucellosis, direct transmission)

Wildlife hosts

- African buffalo as only long-term maintenance host. Can harbor virus asymptomatically
- All cloven-hoofed mammals are essentially susceptible
- Impala: social behavior and herd dynamics can facilitate rapid transmission among individuals
- Kudu: Their movements can aid in the dissemination of the virus across different regions.
- Warthog
- Blue wildebeest

Snotsiekte (malignant catarrhal fever)

High risk-period during wildebeest calving period

Wildlife hosts

- Blue wildebeest
- Black wildebeest

- Sheep typically do not exhibit clinical signs, they can transmit the virus to cattle, resulting in snotsiekte.

Lumpy skin disease:

Wildlife hosts: all of these are identified as carriers for

- African buffalo
- Eland
- Impala
- Giraffe
- Springbok
- Oryx

African horse sickness

African swine fever (direct or via ticks)

Wildlife hosts : (asymptomatic)

- Warthogs
- Bushpigs
- Wild pigs

Bovine tuberculosis

three day stiffness, pink eye (Kerato-conjunctivitis), African horse sickness, heart water, btb, lumpy skin disease, blue tongue

- ➢ Check transmission routes

Questions for Interview: options

Demographic

1. What is your name?
2. What is your occupation?
3. Where do you live?
4. How long have you lived there?
5. Gender

Livestock and Diseases

6. How many livestock do you have?
7. Which livestock do you have?
8. How long have you owned your livestock?
9. Have your animals been affected by any diseases? (*Probe further to know which diseases & symptoms, e.g. abortion)*
10. Do you treat your livestock? *(probe further on what and how treatment is done)*
11. Do you know about xxxxx diseases? *(Maybe show pictures)*

Rewilding Questions

6. What are the wildlife you have seen in this area?
7. Have you seen any changes in the population of xxxx wild animals?
8. Which wild animals interact with your livestock? (*probe on the kind of interactions)*
9. Are there any new wildlife you have seen in recent years that were not here before? *(And vice versa)*
10. What do you think about increasing the population of the current wildlife in this area?
11. Do you think that increasing will affect your livestock? If yes, how?
12. How do you manage the interaction between livestock and wild animals in the area?

13. What do you think about new species bringing in the area?
14. Do you think that introducing new wildlife will affect your livestock? If yes, how?
15. Do you see increased risk for disease transmission between wildlife and domestic animals? And therefore zoonoses? Which diseases and why?

Vet

- Which diseases occur in this area?
- How many animals do you treat with these symptoms each year?
- Vector borne diseases?
- Do people also treat their animals themselves?
- What is the status of acaricide resistance in this area?
- How big of an issue is rabies?
- How many dogs are vaccinated?
- How would you feel about introducing wildlife in this area?
- Do you see increased risk for disease transmission between wildlife and domestic animals?

Healthcare Professionals

- Which diseases and why?
- Which diseases do you treat the most in this area?
- Which zoonotic disease are a problem?
- Do you see many rabies cases?
- What impact in the number zoonotic diseases do you think rewilding will have?

NGOs

- How involved should local communities be in rewilding decisions?
- What could be the benefits of rewilding?
- What are the concerns regarding rewilding?

- What could be the best way of managing diseases when rewilding?
